# Effects of Baduanjin practice on emotional, attention and cognitive function in acupuncturists: protocol for a clinical randomized controlled neuroimaging trial

**DOI:** 10.3389/fpsyg.2024.1340456

**Published:** 2024-04-05

**Authors:** Weiming Luo, Jun Zhou, Xinyue Zhang, Yuke Teng, Siyuan Tao, Nuo Chen, Dan Tong, Peiling Su, Kaijie Ying, Zheng jie Li

**Affiliations:** ^1^Acupuncture and Tuina School, Chengdu University of Traditional Chinese Medicine, Chengdu, China; ^2^Sichuan Health Qigong Management Center, Sichuan Provincial Sports Bureau, Chengdu, China

**Keywords:** acupuncturist, Baduanjin practice, fMRI, neuroimaging, emotion regulation, cognition, attention

## Abstract

**Background:**

In Chinese medicine, the mental focus and emotional stability of acupuncturists are key to optimal clinical outcomes. Many renowned acupuncturists utilize Traditional Chinese Qigong practices to enhance their concentration and emotional regulation abilities. Nevertheless, the existing literature lacks comprehensive evidence addressing this matter.

**Methods:**

This study will enroll 99 acupuncturists and randomly allocate them to one of three groups: Baduanjin, aerobic exercise, or a waiting-list control. The Baduanjin group will undertake 24 weeks of training, with three one-hour sessions weekly. The aerobic group will engage in brisk walking for the same duration and frequency. The control group will not receive any specific training. Assessments of emotion regulation, attention, cognitive functions, finger sensation, and athletic ability will be conducted at baseline (−1 week), mid-intervention (12 weeks), and post-intervention (24 weeks). Additionally, 20 participants from each group will undergo fMRI scans before and after the intervention to explore brain functional and structural changes relating to emotion, attention, cognition, motor skills, and sensory perception.

**Discussion:**

This study aims to contribute valuable insights into the effectiveness of Qigong practice, specifically Baduanjin, in enhancing emotional regulation, attention, and cognitive functions in acupuncturists and to investigate the neuroimaging mechanisms behind these effects.

**Ethics and dissemination:**

Approved by the Sichuan Regional Ethics Review Committee on Traditional Chinese Medicine (No. 2023KL − 118) and adhering to the Declaration of Helsinki. Results will be shared through policy briefs, workshops, peer-reviewed journals, and conferences.

**Clinical trial registration**www.chictr.org.cn, ChiCTR2300076447.

## Background

Acupuncture, now prevalent in 196 countries and regions worldwide, has undergone rapid development and is increasing global acceptance (Liu, 2023). This traditional intervention, deeply rooted in diagnostic assessments, acupoint selection, and adherence to medical guidelines, requires acupuncturists to maintain unwavering focus and emotional calm. Such qualities are crucial for effective treatment, as highlighted in the foundational text of Traditional Chinese Medicine, Huangdi Neijing (Fu and Yang, 2019).

Qigong, a longstanding practice among Chinese acupuncturists, emphasizes the regulation of the body, breath, and mind. Within the diverse array of Qigong forms, Baduanjin emerges as a particularly popular method. Distinguished by its combination of deliberate movements, focused attention, deep breathing, and relaxation, Baduanjin is recognized as a safe and effective form of aerobic exercise. It adheres to the established laws and theories of Qigong in traditional Chinese medicine (Koh, 1982; Fang et al., 2021), with a distinctive focus on harmonizing the mind and body (An et al., 2008).

Recent scientific studies have illuminated the extensive health benefits of Baduanjin across various demographic groups and health conditions. Clinical research has revealed Baduanjin’s potential to bolster physical health, enhance life quality, and improve emotional well-being in healthy individuals (Cheng, 2015; Liao et al., 2015; Zou et al., 2018a). Systematic reviews have demonstrated its effectiveness in reducing symptoms of depression and anxiety in those with physical or mental health challenges (Zou et al., 2018b). Moreover, Baduanjin has shown efficacy in enhancing cognitive functions, including memory and executive function, particularly in older adults with mild cognitive impairment (Tao et al., 2019). Additionally, it has been linked to positive outcomes in various conditions, such as Parkinson’s disease (Liu et al., 2019; Xia et al., 2019), hypertension (Xiong et al., 2015), chronic obstructive pulmonary disease (Liu et al., 2018), type 2 diabetes (Wen et al., 2017), low back pain, and knee osteoarthritis (An et al., 2013; Li et al., 2019).

While many skilled acupuncturists incorporate Traditional Chinese Qigong practices to refine their concentration and emotional regulation, thus improving their acupuncture techniques and effectiveness (Zou et al., 2021), rigorous, evidence-based medicine trials are lacking to substantiate the efficacy of these practices. Moreover, the underlying mechanisms driving these benefits remain largely unexplored.

Neuroimaging technologies, including functional magnetic resonance imaging (fMRI), positron emission tomography (PET), electroencephalography (EEG), and functional near-infrared spectroscopy (fNIRS), have revolutionized the study of neural activity. Among these, fMRI stands out for its convenience, non-invasive nature, and comprehensive brain coverage. This has led to its widespread application in neurology, psychiatry, and cognitive psychology research (Rashid and Calhoun, 2020). Prior neuroimaging studies have identified distinct differences in sensory-motor specialization (Dong et al., 2014, 2015) and emotional control (Dong et al., 2013) between acupuncturists and non-acupuncturists, likely attributable to the unique skills acupuncturists develop in clinical settings.

Given this background, we hypothesize that Baduanjin practice may significantly enhance emotion regulation, cognitive functioning, finger sensation, and motor skills. The aim of this clinical neuroimaging study is to assess the impact of long-term Baduanjin practice, regular aerobic exercise, and a waiting-list control group on these functions. Furthermore, the study intends to explore the neural mechanisms underpinning these effects.

### Objective

(1) To evaluate the effects of Baduanjin exercises on emotion control, attention, and cognitive abilities compared to regular aerobic exercise and a control group on a waiting list.
(2) To visually demonstrate the impact of Baduanjin exercises on brain function related to emotion regulation and its influence on associated networks, including attention, cognitive abilities, motor skills, and sensory functions.

### Hypotheses

Acupuncturist Long-term participation in Qigong will differ significantly from aerobic and waiting exercises in terms of emotional cognition and attention.

*H1*: Long-term participation in Qigong, specifically Baduanjin, is expected to markedly improve acupuncturists’ capabilities in emotion regulation, attention, and cognition.

*H2*: It is also anticipated to cause observable changes in the structure and functional dynamics of specific brain regions.

## Methods and analysis

### Study design

This single-center, parallel randomized controlled clinical trial will enroll 99 eligible subjects from Chengdu University of Traditional Chinese Medicine.

### Participants

The trial aims to recruit 99 participants from the School of Acupuncture and Tuina at Chengdu University of Traditional Chinese Medicine. Recruitment will be facilitated through various channels, including campus radio broadcasts, on-campus advertising, and online platforms.

They will be randomly assigned in a 1:1:1 ratio to the Baduanjin group, the aerobic exercise group, or the waiting-list group.

### Procedure

**Figure 1:**
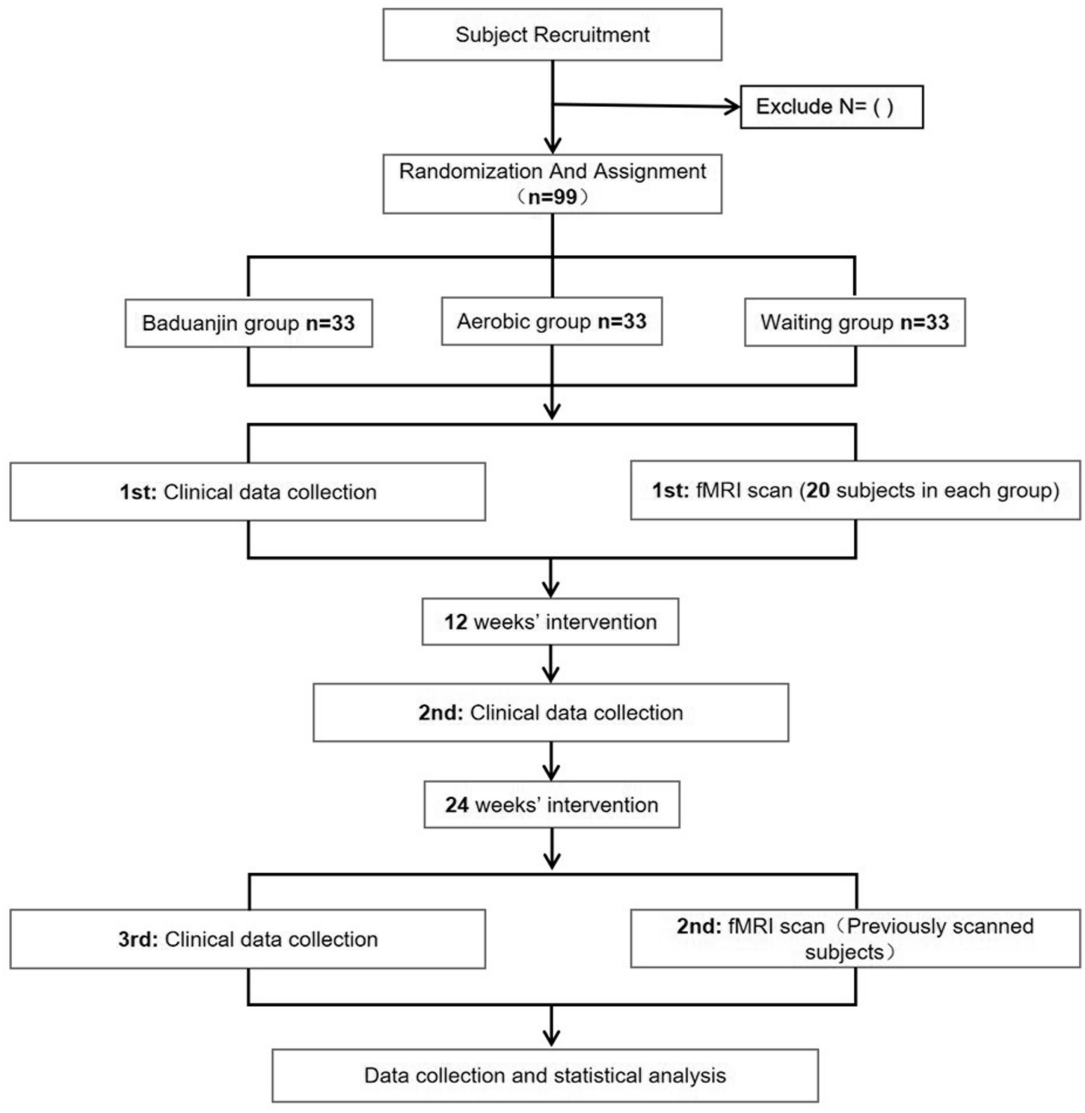
The roadmap of the study process.

The study’s physical and psychological outcomes will be evaluated at three key intervals: one week before the intervention, at 12 weeks (mid-intervention), and at 24 weeks (post-intervention). Additionally, 20 individuals from each group will be selected randomly for pre-and post-intervention fMRI scanning. The study’s process and timeline are depicted in Figure 1 and Table 1.

**Table 1:** Schedule of the trial.

Items			Study process			
			Baseline period	Intervention period (0-24 week)		
Time point			1-week	0-week	12-week	24-week
*Screening and preparation*						
Informed consent			√			
Randomization and allocation			√			
Coaching seminar			√			
*Intervention*						
Baduanjin group				√	√	√
Aerobic exercise group				√	√	√
Waiting group				√	√	√
*Clinical outcomes measurement*	*Primary outcomes*	*Emotional control*				
		SPQ	√		√	√
		IRI	√		√	√
		POMS	√		√	√
		CD-RISC-25	√		√	√
		ECS	√		√	√
		*Attention control*				
		Schulte table software	√		√	√
		*Cognitive function*				
		CFS	√		√	√
	*Secondary outcomes*	*Hand motor function*				
		Hand grip strength	√		√	√
		Finger pinch strength	√		√	√
		Purdue pegboard	√		√	√
		*Hand sensory*				
		Touch-Test	√		√	√
		Two-Point test	√		√	√
		Acupuncture test	√		√	√
*Image scanning*						
fMRI			√			√
*Subject safety*						
Laboratory examination			√			
AEs				√	√	√

This study includes a 1-week advance baseline period and a 24-week intervention period for each participant.

SPQ, the Situational Pain Questionnaire; IRI, the Interpersonal Reactivity Index; POMS, the profile of Mood States; CD-RISC-25, the Chinese version of the Connor-Davidson Mental Toughness Scale; ECS, the Emotional Contagion Scale; CFS, the Cognitive Flexibility Scale; AEs, Adverse events. Laboratory test: including routine examination of blood, urine, and stool, blood biochemical test (including liver and kidney function), and electrocardiogram examination.

#### Inclusion criteria

Subjects will be recruited if they:

Be aged between 18 and 25 years;
Be right-handed;
Provide informed consent;
Be a full-time acupuncture student with completed courses in acupuncture and moxibustion (45 h), meridians and acupoints (84 h), and possess clinical practice experience, as evidenced by passing semester examinations.

#### Exclusion criteria

Subjects will be exclusion if they:

Have chronic pain conditions, a history of head trauma with loss of consciousness, or intellectual disabilities;
Are unable to comply with project requirements;
Suffer from psychiatric, respiratory, cardiovascular, or renal illnesses;
Are pregnant, planning a pregnancy, or lactating;
Have a history of alcohol or substance abuse/dependence;
Show evident organic lesions or severe cranial anatomical asymmetry;
Have contraindications for MRI, such as claustrophobia or non-removable metal in the body;
Engage in long-term regular practice of Qigong, martial arts, dance, aerobics, sparring, or taekwondo.

#### Sample size estimation

This preliminary exploratory clinical randomized controlled neuroimaging trial is informed by similar studies and the minimal sample size guidelines for clinical investigations (Yuen et al., 2021). Accounting for a potential 10% dropout rate, we aim to enroll 33 subjects per group, totaling 99 subjects across three groups. A previous neuroimaging study (Szucs and Ioannidis, 2020) indicates that an imaging sample size of more than 15 per group yields reliable and consistent results. Therefore, 20 subjects from each group will be randomly selected for functional magnetic resonance imaging (fMRI) scans.

### Subjects safety

Prior to the trial, a detailed evaluation of the subjects’ medical histories will be conducted. Routine examinations, including blood, urine, and fecal tests, will be performed before and after the intervention. Electrocardiography tests will also be administered pre-intervention. All adverse events following the intervention will be descriptively documented in the Case Report Form (CRF). Severe adverse events will be closely monitored, managed, and followed up, with immediate reporting to the Ethics Committee within 24 h of occurrence (Harriss et al., 2019).

### Randomization and blinding

Given the nature of this trial, blinding of subjects and exercise coaches is not feasible. The random allocation sequence will be generated using SPSS 26.0 software (SPSS Inc., Chicago, IL, United States) to assign treatments. Allocation will be concealed in sealed, opaque envelopes, opened by the subject management center in sequential order according to subject serial numbers. Subjects will then be allocated to the respective trial groups and adhere to the exercise intervention protocol. A dedicated project manager, separate from the exercise coaches, will oversee the random allocation sequence and the blinding process. The project manager and exercise coaches will not participate in outcome assessments, and likewise, outcome assessors and the statistician will not be involved in subject screening or allocation. The study ensures a strict delineation of roles among researchers, operators, and statisticians.

## Intervention

### Intervention regimen

Previous research (Health Qigong Management Center of General Administration of Sport of China, 2003) indicates that participants in both the Baduanjin and aerobic exercise groups engaged in 60-min sessions, three times weekly, for a duration of 24 weeks. Subjects’ energy expenditure and heart rate (HR) during training will be monitored using a Polar HR monitor (Mio Sports SD).

### Baduanjin group

**Figure 2:**
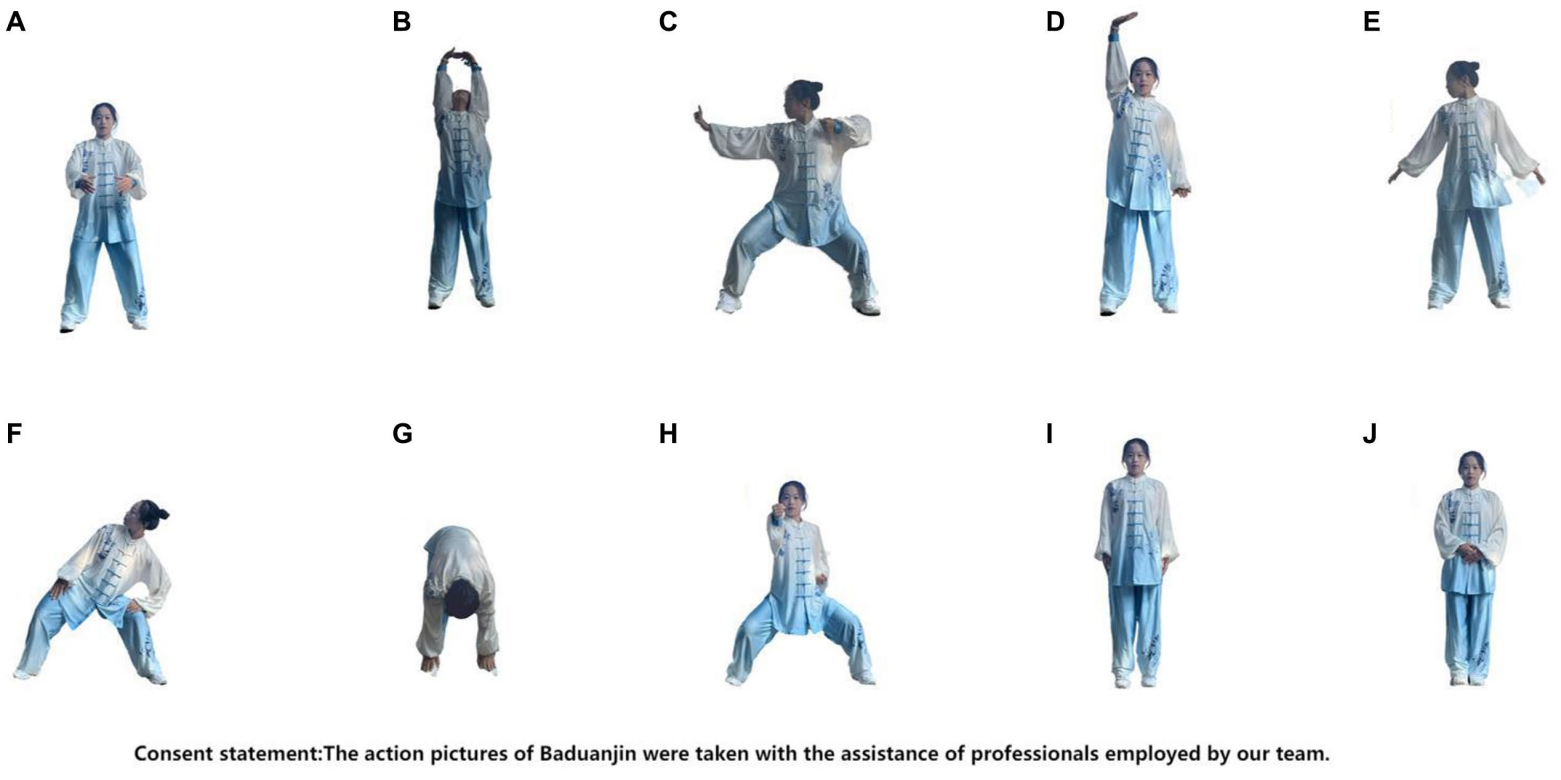
The action pictures of Baduanjin were taken with the assistance of professionals employed by our research team. **(A)** Preparation posture. **(B)** Prop up the sky with hands to regulate the triple energizer. **(C)** Draw a bow on both sides like shooting a vulture. **(D)** Raise a single arm to regulate the spleen (Pi) and stomach (Wei). **(E)** Look back to treat five strains and seven impairments. **(F)** Sway head and buttocks to expel heart (Xin)-fire. **(G)** Pull toes with both hands to reinforce the kidney (Shen) and waist. **(H)** Clench fists and look with eyes wide open to enhance strength and stamina. **(F)** Rise and fall on tiptoes to dispel all diseases. **(J)** Ending posture.

In accordance with the Health Qigong Management Center of General Administration of Sport of China (2003), the Baduanjin group will undergo 24 weeks of training. Each session will last 60 min, comprising a 10-min warm-up, 40 min of Baduanjin exercises, and a 10-min cool-down. This regimen encompasses 10 postures, including preparatory and finishing positions (Figure 2). Supervision and instruction will be provided by a senior social sports instructor with over 5 years of teaching experience. The week preceding the training will feature professional theoretical and movement instruction.

### Aerobic exercise group

Participants in the aerobic exercise group will engage in brisk walking sessions, each lasting 60 min. This includes a 10-min warm-up, 40 min of brisk walking, and a 10-min rest period. Training duration and frequency will mirror those of the Baduanjin group. Professional coaches will guide these sessions, ensuring that exercise intensity remains within 55–75% of the participants’ reserve heart rate, consistent with Baduanjin group guidelines.

### Waiting-list group

The waiting-list group will not participate in any specific exercise regimen. Instead, they will be instructed to maintain their regular activity habits and provide monthly exercise reports.

## Outcome measurements

Outcome measurements will be conducted by trained independent assessors, and all results will be recorded, irrespective of subject completion status. The study timeline, including enrollment, interventions, assessments, and visits, is outlined in Table 1.

### Primary outcome measurement

Primary outcomes focus on attention control, cognitive function, and emotional control, assessed using various tools:

- Schulte Table Software (Prokopenko et al., 2013) for evaluating concentration and attention switching.
- Cognitive Flexibility Scale (CFS) (López et al., 2022) for assessing cognitive flexibility.
- To evaluate the capacity for regulating emotions: Emotional Contagion Scale (ECS) (Doherty, 1997) for measuring susceptibility to others’ emotions.

### Secondary outcome measurement

Secondary outcomes involve additional emotion regulation measures and hand function assessments:

Situational Pain Questionnaire (SPQ) (Stangier et al., 2021): A self-report tool with robust psychometric properties. Interpersonal Reactivity Index (IRI) (Escrivá, 2004): Commonly used for empathy assessment. Profile of Mood States (POMS) (Shahid et al., 2011): Evaluates affective changes during the assessment period. Chinese version of the Connor-Davidson Mental Toughness Scale (CD-RISC-25) (Connor and Davidson, 2003): A brief self-rated tool for quantifying resilience.

Hand motor function is assessed using the Jamar Hand Grip Strength Meter (Wiegert et al., 2022), Jamar Finger Pinch Strength Meter (King, 2013), and Purdue Pegboard Test for manipulative dexterity (Tiffin and Asher, 1948). Hand sensory function is evaluated using the Touch-Test Mono-filament Test (Kaluga et al., 2014), Two-Point Discrimination Sensory Test (Dellon et al., 1987), and a Composite Acupuncture Test developed by the researchers (see Table 2 for details).

**Table 2:** Instructions for the acupuncture test.

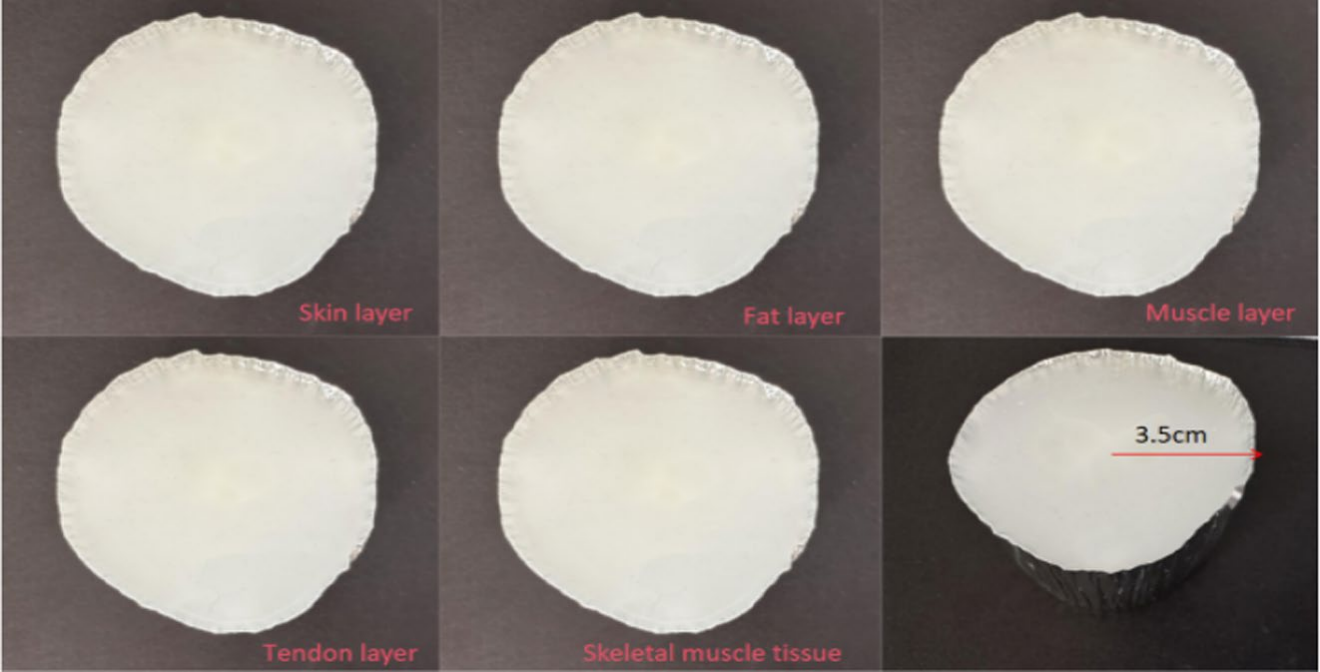	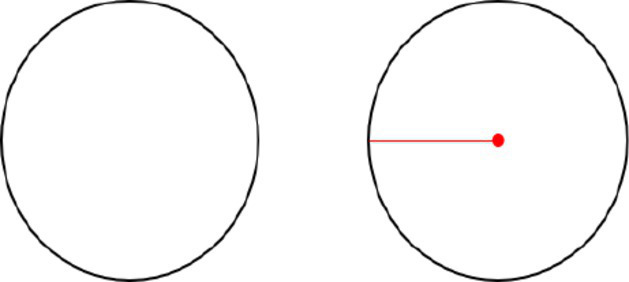

Molds: five human silicone molds that tested by a professional senior acupuncture physician, the hardness of the mold was simulated for the corresponding human skin, fat, muscle, tendon, and skeletal muscle tissue, respectively.

Test items: Sensory test, subjects will acupuncture the center of the mold circle and rank the mold hardness within 15 s in blinding. Accuracy test, the right circular sticker was applied to the mold’s surface, and subjects were instructed to puncture the sticker’s center. The measurement was taken of the separation between the sticker’s pinhole and the circle’s center. Depth test, the subjects needled the mold with the same hardness such as muscle layer, and the distance of the needle body was measured.

### fMRI outcome measurement

MRI data will be acquired using a 3.0-T GE MR750 scanner (GE 3.0 T MR750, Wauwatosa, WI, United States) with an eight-channel phase-array head coil at the University of Electronic Science and Technology of China’s MRI Center.

Scanning parameters include: Structural imaging: Repetition time (TR)/Echo time (TE) = 1900 ms/2.26 ms; slice thickness = 1 mm; 30 slices; matrix size = 128 × 128; field of view (FOV) = 256 × 256 mm. BOLD-fMRI: TR/TE = 2000 ms/30 ms; flip angle = 90°; 30 slices; matrix size = 128 × 128; FOV = 240 × 240 mm; slice thickness = 5 mm; total volume = 240. DTI: TR/TE = 6,800 ms/93 ms; FOV = 240 × 240 mm; matrix size = 128 × 128; slice thickness = 3 mm; two diffusion-weighted sequences with *b* = 1000s/mm^2^ and *b* = 0 across 30 non-collinear directions.

### Statistical methods

A detailed statistical plan, including data requirements and processing methods, will be provided to the statisticians by the research team before data analysis. Statisticians, independent and blinded to the test settings, will execute the analysis as per this plan.

### Behavioral analysis

Data analysis will be conducted by an independent statistician using SPSS version 26.0 (SPSS Inc., Chicago, IL, United States). Two-tailed analyzes will be performed, with statistical significance set at *p* < 0.05.

Intention-to-treat analyzes will be applied wherever possible, complemented by subgroup and sensitivity analyzes to identify potential heterogeneity in results. Continuous variables will be expressed as means ± standard deviations, while categorical variables will be presented as numbers and percentages. The normality of continuous variables will be assessed using the Kolmogorov–Smirnov test. For normally distributed data, within-group differences will be evaluated using paired-sample t-tests, and between-group analyzes will employ Analysis of Variance (ANOVA) and the Kruskal–Wallis test. Non-parametric tests will be used for data with skewed distributions.

### Neuroimaging data analysis

The fMRI data will be conducted pre-processing in accordance with SPM12^1^ and MATLAB 2013b (MathWorks, Inc., Natick, MA, United States).

Data preprocessing will include discarding the first 5 time-points due to magnetic saturation effects, slice timing, head-motion correction, spatial normalization in Montreal Neurological Institute (MNI) space (resampled at 3 mm × 3 mm × 3 mm voxel size), filtering, and spatial smoothing with an 8-mm FWHM Gaussian kernel.

After preprocessing, the amplitude of low-frequency fluctuation (ALFF), fractional ALFF (fALFF), regional homogeneity (ReHo), functional connectivity (FC), and other brain functional parameters will be analyzed. The 3D-T1 data will be processed using voxel-based morphometry in SPM12 and the CAT12 toolbox in MATLAB (Ashburner and Friston, 2000). DTI data will undergo preprocessing and analysis with the Functional Software Library v4.1.9.^2^ Fractional anisotropy (FA) indices and mean diffusivity (MD) maps will be derived after correcting for head motion and eddy currents in DTI images, followed by tensor model fitting. Correlation analyzes will involve Pearson correlation coefficients between clinical and fMRI data, primarily focusing on functional and structural changes in brain regions associated with emotions, attention, motor skills, and sensory perception.

## Discussion

This study represents the first exploration into the patterns and mechanisms associated with Qigong practice among acupuncturists. Its outcomes will contribute initial insights into the effects of Qigong on emotional and attention control while revealing neuroimaging mechanisms unique to acupuncturists. Additionally, these findings will provide empirical support for integrating Qigong into strategies aimed at enhancing acupuncturists’ skills.

### Clinical trial quality control

This study will implement rigorous procedures to mitigate bias and enhance the reliability and reproducibility of the results.

To ensure uniform baseline results among subjects, strict screening and inclusion criteria have been established. Participants must be professionals with completed and verified theoretical and practical training in acupuncture. They should not have engaged in traditional fitness Qigong exercises (such as Baduanjin and Taiji) or mind–body practices (such as yoga and meditation) recently or previously.

For the Baduanjin and aerobic exercise groups, we have defined standardized training protocols, including specific movements, duration, and frequency. Experienced coaches (with over 5 years of practice) will supervise these sessions to guarantee consistency. Training sessions will be video recorded, and random assessments will be conducted to ensure adherence to established standards. The roles of researchers, operators, and statisticians have been distinctly defined, with professional statisticians undergoing specialized training prior to data analysis.

To maintain protocol adherence for all researchers to the study protocol, weekly group meetings will be held for effective communication and continuous learning. After the trial, participants will receive activity credit certificates and reimbursement. Any challenges encountered during the trial will be addressed promptly during these meetings.

### Quality control of imaging tests

In this study, participants aged between 18 and 25 years and who are right-handed will be selected to minimize confounding effects on brain function and structure related to age and handedness. In addition, all neuroimaging scans will be conducted using the same scanner operated by an expert professional.

Before scanning, subjects will receive comprehensive information about the process and environment, along with an emotional assessment. They will be advised to avoid excessive exercise, late nights, and the consumption of alcohol, tobacco, tea, and coffee for 3 days before the scan. During the scan, subjects will wear sponge earplugs and have foam padding around their heads to limit movement. They will be instructed to keep their eyes closed but remain awake. The scanning room will be maintained at 18°C–22°C with humidity above 60% and noise levels below 150 dB.P After the scan, the data will undergo thorough review by professionals, and any data affected by artifacts, signal loss, distortion, or noise interference due to head movement will be excluded from the analysis.

### Limitations

This trial faces several potential limitations. Firstly, due to the nature of the intervention, neither participants nor sports trainers can be blinded. While psychological outcomes are based on self-reports, assessments of hand function and imaging data will be conducted under blinded conditions. Secondly, the study’s single-university setting, a result of funding constraints, may limit the representativeness of the results. Thirdly, although the intervention spans 24 weeks, this duration does not fully capture the long-term effects of Qigong practice on acupuncturists, indicating the need for future multi-center, large-sample randomized controlled studies.

## Ethics statement

The studies involving humans were approved by Sichuan Regional Ethics Review Committee on Traditional Chinese Medicine. The studies were conducted in accordance with the local legislation and institutional requirements. The participants provided their written informed consent to participate in this study. Written informed consent was obtained from the individual(s) for the publication of any potentially identifiable images or data included in this article.

## Author contributions

WL: Writing – original draft, Writing – review & editing. JZ: Writing – original draft, Writing – review & editing. XZ: Methodology, Writing – review & editing. YT: Validation, Writing – review & editing. ST: Investigation, Writing – review & editing. NC: Investigation, Writing – review & editing. DT: Data curation, Writing – review & editing. PS: Supervision, Writing – review & editing. KY: Methodology, Supervision, Writing – review & editing. ZL: Writing – review & editing.

## Footnotes

^1^ http://www.fil.ion.ucl.ac.uk/spm

^2^ http://fsl.fmrib.ox.ac.uk/fsl/fslwiki/TBSS/

## References

[ref1] An B. Dai K. Zhu Z. Wang Y. Hao Y. Tang T. . (2008). Baduanjin alleviates the symptoms of knee osteoarthritis. J. Altern. Complement. Med. 14, 167–174. doi: 10.1089/acm.2007.0600 18315512

[ref2] An B. Wang Y. Jiang X. Lu H. Fang Z. Wang Y. . (2013). Effects of Baduanjin exercise on knee osteoarthritis: a one-year study. Chin. J. Integr. Med. 19, 143–148. doi: 10.1007/s11655-012-1211-y, PMID: 23001463

[ref3] Ashburner J. Friston K. J. (2000). Voxel-based morphometry—the methods. NeuroImage 11, 805–821. doi: 10.1006/nimg.2000.0582, PMID: 10860804

[ref4] Cheng F. K. (2015). Effects of Baduanjin on mental health: a comprehensive review. J. Bodyw. Mov. Ther. 19, 138–149. doi: 10.1016/j.jbmt.2014.11.001, PMID: 25603754

[ref5] Connor K. M. Davidson J. R. T. (2003). Development of a new resilience scale: the Connor-Davidson resilience scale (CD-RISC). Depress. Anxiety 18, 76–82. doi: 10.1002/da.10113 12964174

[ref6] Dellon A. L. Mackinnon S. E. Crosby P. M. (1987). Reliability of two-point discrimination measurements. J. Hand Surg. Am. 12, 693–696. doi: 10.1016/S0363-5023(87)80049-7 3655225

[ref7] Doherty R. W. (1997). The emotional contagion scale: a measure of individual differences. J. Nonverbal Behav. 21, 131–154. doi: 10.1023/A:1024956003661

[ref8] Dong M. Li J. Shi X. Gao S. Fu S. Liu Z. . (2015). Altered baseline brain activity in experts measured by amplitude of low frequency fluctuations (ALFF): a resting state fmri study using expertise model of acupuncturists. Front. Hum. Neurosci. 9:99. doi: 10.3389/fnhum.2015.00099, PMID: 25852511 PMC4365689

[ref9] Dong M. Qin W. Zhao L. Yang X. Yuan K. Zeng F. . (2014). Expertise modulates local regional homogeneity of spontaneous brain activity in the resting brain: An fmri study using the model of skilled acupuncturists. Hum. Brain Mapp. 35, 1074–1084. doi: 10.1002/hbm.22235, PMID: 23633412 PMC6869809

[ref10] Dong M. Zhao L. Yuan K. Zeng F. Sun J. Liu J. . (2013). Length of acupuncture training and structural plastic brain changes in professional acupuncturists. PLoS One 8:e66591. doi: 10.1371/journal.pone.0066591, PMID: 23840505 PMC3686711

[ref11] Escrivá V. M. (2004). La medida de la empatía: análisis del Interpersonal Reactivity Index. Psicothema 16, 255–260.

[ref12] Fang J. Zhang L. Wu F. Ye J. Cai S. Lian X. (2021). The safety of Baduanjin exercise: a systematic review. Evid. Based Complement. Alternat. Med. 2021, 1–11. doi: 10.1155/2021/8867098, PMID: 33552220 PMC7847359

[ref13] Fu J. Yang M.. The yellow Emperor’s classic of medicine — essential questions: translation of Huangdi Neijing Suwen. World Scientific (2019). Singapore

[ref14] Harriss D. J. Mac Sween A. Atkinson G. (2019). Ethical standards in sport and exercise science research: 2020 update. Int. J. Sports Med. 40, 813–817. doi: 10.1055/a-1015-3123 31614381

[ref15] Health Qigong Management Center of General Administration of Sport of China: Health Qigong–Baduanjin. Beijing: People’s Sports Publishing House of China; (2003)

[ref16] Kaluga E. Kostiukow A. Samborski W. Rostkowska E. (2014). Tactile sensitivity on the hands skin in rheumatic patients. Pdia 3, 139–145. doi: 10.5114/pdia.2014.40933, PMID: 25097484 PMC4112261

[ref17] King T. I. (2013). Interinstrument reliability of the Jamar electronic dynamometer and pinch gauge compared with the Jamar hydraulic dynamometer and B & L engineering mechanical pinch gauge. Am. J. Occup. Ther. 67, 480–483. doi: 10.5014/ajot.2013.007351, PMID: 23791323

[ref18] Koh T. C. (1982). Baduanjin-an ancient Chinese exercise. Am. J. Chin. Med. 10, 14–21. doi: 10.1142/S0192415X8200004X, PMID: 7183203

[ref19] Li H. Ge D. Liu S. Zhang W. Wang J. Si J. . (2019). Baduanjin exercise for low back pain: a systematic review and meta-analysis. Complement. Ther. Med. 43, 109–116. doi: 10.1016/j.ctim.2019.01.021, PMID: 30935517

[ref20] Liao Y. Lin Y. Zhang C. Xue X.-L. Mao Q.-X. Zhang Y. . (2015). Intervention effect of Baduanjin exercise on the fatigue state in people with fatigue-predominant subhealth: a cohort study. J. Altern. Complement. Med. 21, 554–562. doi: 10.1089/acm.2014.0395, PMID: 26083663

[ref21] Liu B. (2023). Promoting acupuncture-moxibustion to be world leading discipline. World J. Tradit. Chin. Med. 33:299. doi: 10.1016/j.wjam.2023.05.001

[ref22] Liu J. Chen L. Tu Y. Chen X. Hu K. Tu Y. . (2019). Different exercise modalities relieve pain syndrome in patients with knee osteoarthritis and modulate the dorsolateral prefrontal cortex: a multiple mode MRI study. Brain Behav. Immun. 82, 253–263. doi: 10.1016/j.bbi.2019.08.193, PMID: 31472246

[ref23] Liu S.-J. Ren Z. Wang L. Wei G.-X. Zou L. (2018). Mind–body (Baduanjin) exercise prescription for chronic obstructive pulmonary disease: a systematic review with meta-analysis. Int. J. Environ. Res. Public Health 15:1830. doi: 10.3390/ijerph15091830, PMID: 30149535 PMC6165467

[ref24] López M. B. Arán Filippetti V. Richaud M. C. (2022). Adult executive functioning inventory (ADEXI): factor structure, convergent validity, and reliability of a Spanish adaptation. Appl. Neuropsychol. Adult 29, 1380–1386. doi: 10.1080/23279095.2021.1880408, PMID: 33587681

[ref25] Prokopenko S. V. Mozheyko E. Y. Petrova M. M. Koryagina T. D. Kaskaeva D. S. Chernykh T. V. . (2013). Correction of post-stroke cognitive impairments using computer programs. J. Neurol. Sci. 325, 148–153. doi: 10.1016/j.jns.2012.12.024, PMID: 23312291

[ref26] Rashid B. Calhoun V. (2020). Towards a brain-based predictome of mental illness. Hum. Brain Mapp. 41, 3468–3535. doi: 10.1002/hbm.25013, PMID: 32374075 PMC7375108

[ref27] Shahid A. Wilkinson K. Marcu S. Shapiro C. M. (2011). “Profile of mood states (POMS)” in STOP, THAT and one hundred other sleep scales. eds. Shahid A. Wilkinson K. Marcu S. Shapiro C. M. (New York, NY: Springer New York), 285–286.

[ref28] Stangier U. Schüller J. Brähler E. (2021). Development and validation of a new instrument to measure social pain. Sci. Rep. 11:8283. doi: 10.1038/s41598-021-87351-3, PMID: 33859226 PMC8050222

[ref29] Szucs D. Ioannidis J. P. (2020). Sample size evolution in neuroimaging research: An evaluation of highly-cited studies (1990–2012) and of latest practices (2017–2018) in high-impact journals. NeuroImage 221:117164. doi: 10.1016/j.neuroimage.2020.117164, PMID: 32679253

[ref30] Tao J. Liu J. Chen X. Xia R. Li M. Huang M. . (2019). Mind-body exercise improves cognitive function and modulates the function and structure of the hippocampus and anterior cingulate cortex in patients with mild cognitive impairment. Neuroimage 23:101834. doi: 10.1016/j.nicl.2019.101834, PMID: 31128522 PMC6535682

[ref31] Tiffin J. Asher E. J. (1948). The Purdue pegboard: norms and studies of reliability and validity. J. Appl. Psychol. 32, 234–247. doi: 10.1037/h0061266, PMID: 18867059

[ref32] Wen J. Lin T. Cai Y. Chen Q. Chen Y. Ren Y. . (2017). Baduanjin exercise for type 2 diabetes mellitus: a systematic review and Meta-analysis of randomized controlled trials. Evid. Based Complement. Alternat. Med. 2017, 1–14. doi: 10.1155/2017/8378219, PMID: 29234435 PMC5671720

[ref33] Wiegert E. V. M. Da Silva N. F. De Oliveira L. C. Calixto-Lima L. (2022). Reference values for handgrip strength and their association with survival in patients with incurable cancer. Eur. J. Clin. Nutr. 76, 93–102. doi: 10.1038/s41430-021-00921-6, PMID: 33911207

[ref34] Xia R. Qiu P. Lin H. Ye B. Wan M. Li M. . (2019). The effect of traditional Chinese mind-body exercise (Baduanjin) and brisk walking on the dorsal attention network in older adults with mild cognitive impairment. Front. Psychol. 10:2075. doi: 10.3389/fpsyg.2019.02075, PMID: 31551895 PMC6748214

[ref35] Xiong X. Wang P. Li S. Zhang Y. Li X. (2015). Effect of Baduanjin exercise for hypertension: a systematic review and meta-analysis of randomized controlled trials. Maturitas 80, 370–378. doi: 10.1016/j.maturitas.2015.01.002, PMID: 25636242

[ref36] Yuen M. Ouyang H. X. Miller T. Pang M. Y. C. (2021). Baduanjin qigong improves balance, leg strength, and mobility in individuals with chronic stroke: a randomized controlled study. Neurorehabil. Neural Repair 35, 444–456. doi: 10.1177/15459683211005020, PMID: 33825587

[ref37] Zou L. Pan Z. Yeung A. Talwar S. Wang C. Liu Y. . (2018a). A review study on the beneficial effects of Baduanjin. J. Altern. Complement. Med. 24, 324–335. doi: 10.1089/acm.2017.0241, PMID: 29227709

[ref38] Zou D. Wang H. Wu S. Li Q. Yu Y. Li J. . (2021). Discussion on the teaching of "practicing acupuncture" by famous acupuncturists. Guid. J. Trad. Chin. Med. Pharmacol. 27, 209–212. doi: 10.13862/j.cnki.cn43-1446/r.2021.12.049

[ref39] Zou L. Yeung A. Quan X. Hui S. S.-C. Hu X. Chan J. S. M. . (2018b). Mindfulness-based Baduanjin exercise for depression and anxiety in people with physical or mental illnesses: a systematic review and meta-analysis. Int. J. Environ. Res. Public Health 15:321. doi: 10.3390/ijerph15020321, PMID: 29439556 PMC5858390

